# First studies showing high temephos resistance in *Anopheles labranchiae* (*Diptera*: *Culicidae)* from Tunisia

**DOI:** 10.4314/ahs.v18i1.7

**Published:** 2018-03

**Authors:** Ahmed Tabbabi, Jabeur Daaboub

**Affiliations:** Department of Hygiene and Environmental Protection, Ministry of Public Health, Tunis, Tunisia

**Keywords:** *Anopheles labranchiae*, temephos insecticide, Tunisia

## Abstract

**Background:**

Despite the public health importance of *Anopheles (An.) labranchiae*, their resistance status to temephos insecticide has not, to our knowledge, been explored.

**Objectives:**

The present study was carried out to determine the temephos resistance status of field populations of *An. labranchiae* from Tunisia.

**Methods:**

Six field populations of *An. labranchiae* were collected as larvae from breeding sites of Northern and Central Tunisia. All the tests were carried out according to the WHO method.

**Results:**

Results reported that the majority of field populations showed low and medium resistance ratios (6.2<RR50< 29.8) to temephos insecticide tested except for the strain # 1 which had interestingly a very high resistance with RR50 of 624 never detected in Tunisia and North Africa even on other species of mosquitoes

**Conclusion:**

The resistance ratios of this species were higher than recorded in other countries. Biochemical and molecular studies would be of great importance to identify the mechanisms involved in the recorded resistance to temephos.

## Introduction

In Tunisia, The last registered case of autochthonous malaria was reported in 1979. Since then a vector control involves killing the larvae of the main malaria vectors, *An. labranchiae* (Falleroni, 1926), through the judicious use of environmentally safe insecticides was reported to prevent re-establishment of malaria transmission and to control other vector borne diseases^1^–^4^. Mosquito control with the use of insecticides was faced with the challenges of insecticide resistance in malaria vectors, community refusal, their high cost, operational difficulties, and environmental concern. In view of this, integrated vector control strategies with the use of larvivorous fishes such as Gambusia (*G. affinis*) as biological control agents were used in controlling mosquito breeding in different types of habitat places.

In Tunisia, an intensive control program was carried out in the framework of the National Program for the Eradication of Malaria during the 60s and 70s against malaria vectors mainly through the use of DDT. The pathology disappeared from the country since 1979. Since then, other insecticides including temephos organophosphate were used for prevention and control of vector-borne diseases^2^–^5^. Two important organophosphate resistance mechanisms exhibited by insects are metabolic-based resistance^3^,^5^ including three groups of detoxifying enzymes: mono-oxygenases, esterases, and glutathione S-transferases and target site insensitivity including acethylcholinesterases resistance^3^,^5^.

Therefore, periodic evaluation of insecticide susceptibility is a necessary part of good pest management practice. Despite the public health importance of *An. labranchiae*, their susceptibility tests to temephos have not, to our knowledge, been explored. A good understanding of their resistance status could improve vector control implementation through targeted strategies.

Temephos insecticide is considered by World Health Organization^6^ as a suitable and safe mosquito larvicide that can be used even in drinking water for controlling of the most mosquito vectors. The temephos discriminating dosage, established by WHO, for the genus *Anopheles* is 0.25 mg/L. Afterward, it was stated locally for a limited number of species^7^. For *An. Hyrcanus*, this dose was set at 0.025 mg/L and it was 0.625 mg/L for *An. sacharov* that belongs to the same complex as *An. labranchiae*.

The present study was carried out to determine the temephos resistance status of field populations of *An. labranchiae* from Tunisia. This study will also serve as reference data which could be applied in countries around Mediterranean sea, particularly Morocco, Algeria, Italy, and France where *An. labranchiae* is present and could constitute a risk for malaria transmission^8^–^11^.

## Materials and methods

### Mosquito strains and study areas

**Figure 1:**
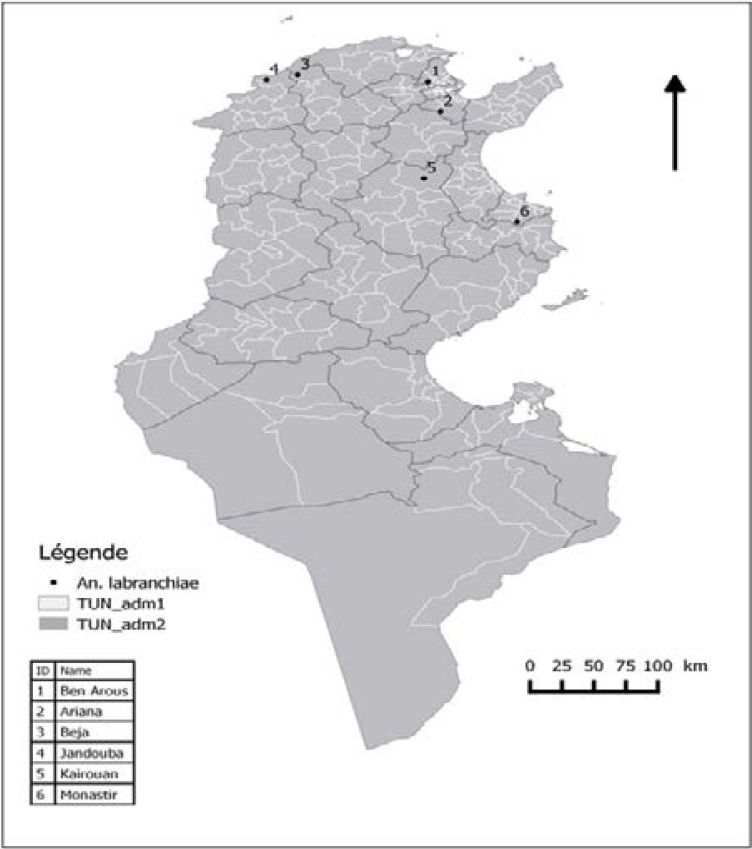
Geographic origin of Tunisian populations

Six field populations of *An. labranchiae* were collected as larvae from breeding sites of Northern and Central Tunisia (Figure 1). A sensitive strain was used as reference to compare the level of resistance with resistant strains. It should be noted that general characteristics of study areas including insecticides usage is given in Table 1. Data was collected according to the ministry of health and during individual interviews with the collection sites residents. The larvae were transferred to the laboratory in the original water. The larvae were putted directly in clean standing water in the laboratory and fed daily with ground food mixture. Larvae were used for bioassays and eggs were reared to larvae under laboratory conditions. Larvae from eggs were used for toxicological tests if the number of collected larvae was not enough.

**Table 1:** Geographic origin of Tunisian populations of *An. labranchiae*, breeding site characteristics, and insecticide control

Code	Governorate	Breeding sites	Date of collection	Mosquito control (used insecticides)	Agricultural pest control
1	Ben Arous	Ditch	Oct. 2016	Occasional (P,D)	Yes
2	Ariana	River	Oct. 2016	Rare (C,P)	Yes
3	Beja	Water pond	Nov. 2016	None	Yes
4	Jandouba	River	Nov. 2016	Occasional (P)	Yes
5	Kairouan	Ditch	Oct. 2016	None	Yes
6	Monastir	Water pond	Oct. 2016	Rare (C,F)	Yes

C : Chlorpyrifos; F : Fenitrithion; P : Permethrin; D : Deltamethrin

### Chemicals insecticides and synergists

The insecticides tested were the organophosphates temephos (9l%o; American Cyanamid, Princeton, NJ), and the carbamate propoxur (997o; Mobay). Two synergists were used to help detect detoxification enzymes involved in resistance: S, S, S {ributyl phosphorothioate (DEF), an esterase inhibitor, and piperonyl butoxide (Pb), an inhibitor of mixed function oxidases.

### Resistance tests

The susceptibility to temephos insecticide was carried out on late 3^rd^ or early 4^th^ instar larvae of the filed populations. Third- and fourth-instar larvae were morphologically identified using the standardized key for the mosquitoes of Mediterranean Africa^12^. The bioassays followed the recommended experimental protocol standardized by the WHO^13^. Five concentrations were used for each insecticide. Triplicate of 20 larvae were employed for each concentration and mortality was noted after 24 hours. Triplicate of control were used and exposed to alcohol. The bioassay was cancelled if mortality exceeded 10%.

### Data analysis

We used log probit analysis of Raymond^14^, based on Finney^15^ which provides LCs, slope for each mortality line, parallelism between different mortality lines and resistance ratios with 95% confidence intervals to analyze mortality data.

## Results

### Insecticides resistance

As showed in Table 2, the majority of field populations showed low and medium resistance ratios (6.2<RR50<29.8) to temephos insecticide tested except for the strain # 1 which had interestingly a very high resistance with RR50 of 624 never detected in Tunisia and North Africa even on other species of mosquitoes. Slopes values of studied populations were compared with sensitive strain and showed lower heterogeneity.

**Table 2:** Temephos resistance characteristics of Tunisian *An. labranchiae* in presence and absence of synergists DEF and Pb

Population	Temephos			Temephos +DEF					Temephos +Pb				

	LC_50_ in µg/l (a)	Slope ± SE	RR_50_ (a)	LC_50_ in µg/l (a)	Slope ± SE	RR_50_ (a)	SR_50_ (a)	RSR	LC_50_ in µg/l (a)	Slope ± SE	RR_50_ (a)	SR_50_ (a)	RS R
**Sensitive** **strain**	0.9 (0.1–1.2)	2.18 ± 0.31	-	0.41 (0.22–0.57)	3.78 ± 0.59	-	2.1 (1.8–3.0)	-	0.56 (0.3–1.1)	0.88 ± 0.17	-	1.60 (1.33–2.12)	-
**1-Ben** **Arous**	561.6 (480.4–621.8)	2.395 ± 0.28	624 (595.5–659.4)	120.49 (114–135.2)	2.45 ± 0.45	263.87 (222.5–285.4)	4.66 (6.18–10.3)	2.3 6	489.8 (401.1–590.4)	1.99 * ± 0.33	874.64 (799.6–320.9)	1.14 (0.12–1.40)	0.71
**2-Ariana**	9.2 (7.4–12.3)	3.58 * ± 0.16	10.2 (8.7–16.3)	7.2 (5.7–9.3)	1.18 ± 0.22	17.5 (13.5–18.7)	1.2 (0.9–2.4)	0.5 8	10.3 (8.7–12.9)	3.01 ± 0.21	18.39 (17.4–19.4)	0.89 (0.67–1.35)	0.55
**3-Beja**	5.6 (4.1–7.0)	1.55 ± 0.05	6.2 (3.0–9.7)	4.3 (3.9–5.1)	1.97 ± 0.23	10.4 (8.7–13.0)	1.3 (0.8–1.8)	0.5 9	6.9 (5.19–8.12)	1.08 ± 0.24	12.32 (11.52–13.52)	0.81 (0.57–2.1)	0.50
**4-Jandouba**	22.9 (18.3–26.8)	2.74 ± 0.35	25.4 (19.8–28.4)	5.76 (4.22–7.67)	0.90 ± 0.18	14.0 (11.9–17.4)	3.9 (2.35–4.05)	1.8 1	12.9 (10.4–14.6)	1.59 * ± 0.20	23.03 (22.6–24.9)	1.77 (0.98–2.7)	1.1
**5-Kairouan**	12.4 (8.2–14.3)	2.11 * ± 0.22	13.7 (10.5–22.2)	10.2 (8.2–11.9)	1.80 ± 0.13	24.8 (20.5–28.7)	1.2 (1.9–2.7)	0.5 5	15.3 (12.4–18.7)	3.12 ± 0.27	27.32 (25.5–33.5)	0.81 (0.55–1.44)	0.50
**6-Monastir**	13.2 (10.4–16.5)	2.59 * ± 0.29	14.6 (11.9–18.2)	0.9 (0.77–1.3)	1.98 ± 0.34	2.2 (1.5–4.7)	14.6 (13.9–15.6)	6.6 3	8.3 (5.7–10.9)	3.45 ± 0.33	14.82 (13.3–16.2)	1.59 (0.99–1.98)	0.98

(a), 95% CI;

* The log dose-probit mortality response is parallel to that of sensitive strain

RR_50_, resistance ratio at LC_50_ (RR_50_=LC_50_ of the population considered/LC_50_ of sensitive strain); SR_50_, synergism ratio (LC_50_ observed in absence of synergist/LC_50_ observed in presence of synergist). RR and SR considered significant (P<0.05) if their 95%CI did not include the value 1; RSR, relative synergism ratio (RR for insecticide alone / RR for insecticide plus synergist).

### Synergism tests

Our results of bioassays with synergists (Table 2) showed that the synergism ratio (SR) in all *An. labranchiae* populations tested using DEF was low (SR<4), except two field populations (# 1 and 6) where SR value was 4.66 and 14.6, respectively. In fact, we found that the DEF synergist reduced resistance to temephos in the two populations, where the resistance ratios were reduced from 624 to 263.87 and 14.6 to 2.2 at LD_50_ in samples # 1 and 6, respectively. These decrease of resistance level confirmed the important role played by esterases on the enzymatic detoxification of these two strains. The results with Pb indicated that oxydases were not involved in temephos resistance of *An. labranchiae*, as detected by the lower SR values in all strains (0.81<SR<1.77).

### Cross-resistance Temephos/Propoxur

We found that mortality caused by propoxur varied from 2% in samples # 1 showing the highest resistance levels to studied temephos insecticide (624 at LC_50_) to 73% in sample # 3 showing the lowest resistance (6.2 at LC_50_). A strong correlation between the mortality due to propoxur and the LC_50_ of temephos (Spearman rank correlation, (r) = -0.72 (P<0.01)) was registered. These results indicated that the insensitive AChE 1 had a major role in the recorded resistance to temephos.

## Discussion

The finding that the majority of field populations showed low, moderate, and high resistance ratios (6.2<RR50<624) to temephos are similar to those obtained by previous studies realized in Tunisia with other mosquitoes species^4^,^16^,^17^ but never on *Anopheles* mosquitoes. Ben Cheilkh et al^4^ showed that field populations of *Culex pipiens* larvae from Tunisia were less resistant to temephos not exceeding 10-folds. Recently, other authors^18^ from the same country reported high resistance with RR50 of 440-folds. Our finding is surprising because temephos insecticide was not the principle larvicide used in Tunisia for successful interruption of autochthonous malaria transmission. These findings should be confirmed by more studies for temephos insecticide on more larval population collected from the same areas.

Resistance of *An. labranchiae* to temephos has previously studied in Morocco country^1^. Authors found low resistance to this larvicide in sentinel site. Study on *An. stephensi* in Oman country^19^ showed moderate resistance to temephos. In India, This species has been reported susceptible^20^,^21^ with LC_50_ range of 0.008–0.015. Despite the minor use of temephos insecticide in last years but study areas are known by their agricultural activities and farmers use frequently organophosphate insecticides to control pests. These activities can affect the susceptibility status of *An. labranchiae* that's why integrated vector management in public health must take into consideration the integrated pest management in agriculture as a priority to limit vector/pests resistance to pesticides.

Our results showed that all tested populations are resistant to temephos despite there are no national program based on temephos insecticide to control this species. In fact, the department of hygiene and environmental protection gives priority to biological (larvivorous fishes) and environmental control. Knowing that waters are the mosquitoes breeding habitat and there are many sources of water contamination, including naturally occurring chemicals and minerals, this species may have developed resistance to these compounds to save itself.

Our results showed that esterases were not associated with resistance in the majority of field populations tested (r = −0.009, p > 0.05). Similar results were found by Selvi et al^22^ in larvae and adults of different species mosquitoes. On the other hand, a strong correlation between esterase activity and resistance to temephos were reported by many authors in Tunisia and other countries^23^–^26^. Any correlation between oxidases activity and RR50 values of temephos obtained from bioassay (r = 0.190, p > 0.05) were detected in our findings. Similar results were found by Paeporn et al^27^. However, The CYP450 enzyme was associated with temephos resistance in previous studies of Ben Cheikh et al^4^. and Bisset et al^28^.

A strong correlation between the mortality due to propoxur and the LC_50_ of temephos (Spearman rank correlation, (r) = -0.72 (P<0.01)) was registered indicating that insensitive AChE 1 play a clear role in temephos resistance as reported by Macoris et al^28^ and Saelim et al^30^. It should be noted that AChE 1 has been well documented as a resistance mechanism of organophosphate and carbamate insecticides in mosquito populations by Hemingway et al^30^. Raymond et al^32^ and Cui et al^33^.

## Conclusion

Our study shows for the first time in Tunisia the resistance status of *An. labranchiae* to temephos insecticide. The resistance ratios of this species were higher than recorded in other countries. Biochemical and molecular studies would be of great importance to identify the mechanisms involved in resistance to temephos.

## References

[1] Faraj C, Adlaoui E, Ouahabi S, Rhajaoui M, Fontenille D, Lyagoubi M (2009). Entomological investigations in the region of the last malaria focus in Morocco. Acta Tropical.

[2] Kooli J, Rhaiem A (1989). Sensibilité des larves de moustiques aux insecticides dans la région de Tunis en 1984. Arch Inst Pasteur Tunis.

[3] Ben Cheikh H, Marrakchi M, Pasteur N (1995). Mise en évidence d'une très forte résistance au chlorpyrifos et à la perméthrine chez les moustiques *Culex pipiens* de Tunisie. Arch Inst Pasteur Tunis.

[4] Ben Cheikh H, Haouas-Ben Ali Z, Marquine M, Pasteur N (1998). Resistance to organophosphorus and pyrethroid insecticides in *Culex pipiens* (*Diptera: Culicidae*) from Tunisia. J Med Entomol.

[5] Pasteur N, Marquine M, Ben Cheikh H, Bernard C, Bourguet D (1999). A new mechanism conferring unprecedented high resistance to chlorpyrifos in *Culex pipiens* (*Diptera : Culicidae*). J Med Entomol.

[6] WHO (2006). Pesticides and their application for the control of vectors and pests of public health importance.

[7] WHO (1992). “Vector resistance to pesticides,” 15th report of the WHO Expert Committee on Vector Biology and Control, Technical Report Series 818.

[8] Romi R (1999). *Anopheles labranchiae*, an important malaria vector in Italy, and other potential malaria vectors in Southern Europe. European Mosquito Bulletin.

[9] Toty C, Barré H, Le Goff G, Larget-Thiéry I, Rahola N, Couret D, Fontenille D (2010). Malaria risk in Corsica, former hot spot of malaria in France. Malaria Journal.

[10] Chahed M, Bouratbine A, Krida G, Ben Hamida A (2001). Réceptivité de la Tunisie au paludisme après son eradication: Analyse de la situation pour une adéquation de la surveillance. Bulletin de la Société de Pathologie Exotique.

[11] Hammadi D, Boubidi SC, Chaib SE, Saber A, Khechache Y, Gasmi M, Harrat Z (2009). Le paludisme au Sahara algérien. Bulletin de la Societe de Pathologie Exotique.

[12] Brunhes J, Rhaim A, Geoffroy B, Angel G, Hervy JP (1999). Les moustiques de l'Afrique méditerranéenne CDROM d'identification et d'enseignement, Edition.

[13] Raymond M, Fournier D, Bride JM, Cuany A, Bergé J, Magnin M, Pasteur N (1986). Identification of resistance mechanisms in *Culex pipiens*(*Diptera: Culicidae*) from southern France: insensitive acetylchlinesterase and detoxifying oxidases. J Econ Entomol.

[14] Raymond M, Prato G, Ratsira D (1993). Analysis of mortality assays displaying quantal response. Praxeme (Licence No. L93019).

[15] Finney DJ (1971). Probit analysis.

[16] Daaboub J, Tabbabi A, Lamari A, Feriani M, Boubaker C (2017). Temephos Resistance in Three Populations of Culex pipiens Collected from Three Districts of Southern Tunisia and Its Significance for the Resistance Mechanism. Vector Biol J.

[17] Tabbabi A, Daaboub J, Lamari A, Ben Cheikh R, Ben Jha I, Ben Cheikh H (2017). Susceptibility of different geographical strains of *culex pipiens* (*diptera: culicidae*) to temephos in grand Tunis area of Tunisia. International Journal of Multidisciplinary and Development.

[18] Daaboub J, Tabbabi A, Lamari A, Ben Cheikh R, Ben Haj Ayed A, Ben Cheikh H (2017). Susceptibility of field-collected mosquitoes (*Culex pipiens*) in Northern Tunisia to temephos, an organophosphate insecticide. Asian Pac J Health Sci.

[19] Parvez SD, Al-Wahaibi SS (2003). Comparison of three larviciding options for malaria vector control. Eastern Mediterr Health J.

[20] Tikar SN, Mendki MJ, Sharma AK, Sukumaran D, Veer V, Parashar BD (2011). Resistance status of the malaria vector mosquitoes, *Anopheles stephensi* and *Anopheles subpictus* towards adulticides and larvicides in arid and semi-arid areas of India. J Insect Sci.

[21] Singh PK, Mittal PK, Kumar G, Dhiman RC (2014). Insecticide susceptibility status of *Aedes aegypti* and *Anopheles stephensi* larvae against temephos in Delhi, India. Int J Mosq Res.

[22] Selvi S (2009). Comparison of esterases between life stages and sexes of resistant and susceptible strains of vector mosquitoes.

[23] Kao LR, Motoyama N, Dauterman WC (1985). The purification and characterization of esterases from insecticide resistant and susceptible houseflies. Pesticide Biochemistry and Physiology.

[24] Yan PC, Sudderuddin KI (1978). Toxicology studies of insecticides on *Culex quinquefasciatus* (Say) and *Aedes aegypti* (L.). Southeast Asian Journal of Tropical Medicine and Public Health.

[25] Chen CD, Nazni WA, Lee HL, Seleena B, Sofian-Azirun M (2008). Biochemical detection of temephos resistance in *Aedes* ( *Stegomyia*) aegypti (*Linnaeus*) from dengue-endemic areas of Selangor State, Malaysia. Proceeding of the 3^rd^ ASEAN Congress of Tropical Medicine and Parasitology.

[26] Ben Cheikh R, Berticat C, Berthomieu A, Ben Cheikh H, Weill M (2008). Characterization of a novel high-activityesterase in Tunisian populations of *Culex pipiens*. J Econ Entomol.

[27] Paeporn P, Komalamisra N, Deesin V, Rongsriyam Y, Eshita Y, Thongrungkiat S (2003). Temephos resistance in two forms of Aedes aegypti and its significance for the resistance mechanism. Southeast Asian Journal of Tropical Medicine and Public Health.

[28] Bisset JA, Rodriguez MM, Diaz C, Soca A (2000). Course of insecticide resistance in *Culex quinquefasciatus* (*Diptera*: *Culicidae*) in a region of La Habana. Rev Cubana Med Trop.

[29] Macoris L, Andrighetti MT, Takaku L, Glasser CM, Garbeloto VC, Bracco JE (2003). Resistance of *Aedes aegypti* from the State of Sao Paulo, Brazil to organophosphates insecticides. Memorias do Instituto Oswaldo Cruz.

[30] Saelim V, Brogdon WG, Rojanapremsuk J, Suvannadabba S, Pandii W, Jones JW, Sithiprasasna R (2005). Bottle and biochemical assays on temephos resistance in *Aedes aegypti* in Thailand. Southeast Asian Journal of Tropical Medicine and Public Health.

[31] Hemingway J, Smith C, Jayawardena KG, Herath PRJ (1986). Field and laboratory detection of the altered acetylcholinesterase resistance genes which confer organophosphate and carbamate resistance in mosquitoes (Diptera: Culicidae). Bulletin of Entomological Research.

[32] Raymond M, Fournier D, Berge J, Cuany A, Bride JM, Pasteur N (1985). Single mosquito test to determine genotypes with an acetylcholinesterase insensitive to inhibition to propoxur insecticide. Journal of American Mosquito Control Association.

[33] Cui F, Raymond M, Berthomieu A, Alout H, Weill M, Qiao CL (2006). Recent emergence of insensitive acetylcholinesterase in Chinese populations of *Culex pipiens* (*Diptera: Culicidae*). Journal of Medical Entomology.

